# Factors associated with work engagement dimensions among hospital nurses in Beijing: a cross-sectional study

**DOI:** 10.3389/fpsyg.2026.1817637

**Published:** 2026-06-25

**Authors:** Zixuan Yan, Linghui Duan, Zhuqing Zhang, Shouyang Zhang, Gangqiang Wang, Wenyu Zhang

**Affiliations:** 1 Department of Senior Citizens Welfare, China Civil Affairs University, Beijing, China; 2 Department of Gastroenterology, The Seventh Medical Center of Chinese PLA General Hospital, Beijing, China; 3 Department of Oncology, The Seventh Medical Center of Chinese PLA General Hospital, Beijing, China; 4 Department of Rehabilitation, The Seventh Medical Center of Chinese PLA General Hospital, Beijing, China; 5 School of Nursing, Capital Medical University, Beijing, China

**Keywords:** family harmony, nurses, role boundaries, work engagement, workload

## Abstract

**Background:**

Work engagement is closely related to nurses’ well-being and workforce stability. This study described work engagement among hospital nurses in Beijing and examined demographic, family-related, and workload-related factors associated with dedication, vitality, and absorption.

**Methods:**

A cross-sectional survey was conducted among clinical nurses from nine hospitals in Beijing, China, between May 2023 and October 2024. Eligible nurses completed an anonymous online questionnaire. Work engagement was measured using the Utrecht Work Engagement Scale (UWES-17). Item-average scores for dedication, vitality, and absorption were analyzed as continuous outcomes. Generalized estimating equations were used to account for within-hospital clustering, and variables with *p* < 0.05 in univariable analyses were entered into multivariable models.

**Results:**

Of 2,040 eligible nurses invited, 2,020 returned questionnaires and 2,017 valid responses were included (valid response rate: 98.9%). The mean overall work engagement item-average score was 4.42 ± 1.25; dedication had the highest mean score (4.72 ± 1.27), followed by vitality (4.37 ± 1.29) and absorption (4.22 ± 1.30). Poorer perceived family harmony was consistently associated with lower engagement across all three dimensions. Higher perceived workload, repeated ≥12-h workdays, and undertaking work outside formal duties and responsibilities were also associated with lower engagement across dimensions. Older age, operating-room placement, higher income, and chief nurse title were generally associated with higher engagement.

**Conclusion:**

Work engagement among nurses in Beijing was associated with both family context and work demands. Poorer family harmony and heavier workload were the most consistent correlates of lower dedication, vitality, and absorption. Reducing avoidable workload, clarifying role boundaries, and supporting nurses’ family lives may help sustain work engagement.

## Introduction

Work engagement is a persistent, positive affective-motivational state comprising vigor, dedication, and absorption (Bargagliotti, 2012). In nursing, higher work engagement has been associated with better quality of care, stronger job satisfaction, lower turnover intention, and more favorable workforce outcomes (Bargagliotti, 2012; Keyko et al., 2016; García-Sierra et al., 2016). This makes work engagement an important indicator not only of individual well-being but also of workforce stability and service performance. In China, where the nursing workforce has expanded rapidly but staffing pressure remains substantial, engagement continues to be a salient management issue (Zhang et al., 2023). Recent evidence further suggests that work engagement is closely linked to turnover intention and occupational well-being among registered nurses, underscoring the need to identify the factors that may sustain or erode it in everyday clinical practice (BowenXue et al., 2024; Yildiz and Yildiz, 2022).

The Job Demands-Resources model provides a useful framework for examining these factors (Bakker and Demerouti, 2017). According to this model, job demands consume energy and may weaken engagement, whereas job and personal resources support motivation and help employees remain psychologically invested in their work (Bakker et al., 2014). Among nurses, workload is one of the most consistently studied demands and has been linked to burnout, strain, and diminished work-related well-being (Dall'Ora et al., 2020; Miller and Hemberg, 2023). Another demand that has received less direct attention is role boundary ambiguity, especially the expectation that nurses undertake tasks outside their core professional responsibilities. Such extra-role assignments may create role strain, blur professional boundaries, and reduce the psychological energy available for engaged work (Tarrant and Sabo, 2010). At the same time, although JD-R research has traditionally emphasized organizational resources such as leadership, autonomy, and team support, resources located outside the workplace also matter (Bakker and Demerouti, 2017; ten Brummelhuis and Bakker, 2012). In the Chinese cultural context, family harmony has long been viewed as a foundation of individual adjustment and resilience (Hwang, 2012; Bedford and Yeh, 2019). From the perspective of Conservation of Resources theory, harmonious family relationships may function as a personal resource that replenishes emotional energy and buffers work strain (Hobfoll, 1989). Research on work-family enrichment also suggests that positive family experiences can enhance functioning in the work domain (Greenhaus and Powell, 2006; McNall et al., 2010).

Against this background, the present study focuses on three factors that may be especially relevant to nurses’ work engagement: family harmony, workload, and role boundaries. We propose that higher family harmony is associated with higher work engagement, whereas heavier workload and more frequent non-duty work are associated with lower work engagement. By bringing together a family-domain resource and two forms of work demand within a single analytical framework, this study extends the usual scope of nursing engagement research, which has more often concentrated on organizational variables alone (Bakker and Demerouti, 2017; ten Brummelhuis and Bakker, 2012). This broader perspective is particularly relevant for understanding nurses’ work experiences in contemporary China, where professional demands and family responsibilities often intersect.

Using a cross-sectional survey of clinical nurses in Beijing, this study aimed to describe the current level of work engagement and to examine whether family harmony, workload, and role boundary clarity were independently associated with engagement. The findings are expected to provide empirical evidence for more targeted strategies to support nurse retention and strengthen the nursing workforce.

## Methods

### Study design and participants

This cross-sectional study investigated clinical nurses working in hospitals across Beijing, China. Data were collected between May 2023 and October 2024. The inclusion criteria were: (1) holding a valid nursing license issued by the People’s Republic of China, (2) currently engaged in clinical nursing duties at the time of data collection, and (García-Sierra et al., 2016) having at least 1 year of clinical experience. Nurses who were on extended leave (e.g., maternity leave, sick leave exceeding 3 months), who held purely administrative positions with no clinical responsibilities, or who were student interns were excluded. Participation was voluntary and anonymous.

Participants were recruited through departmental nursing managers at each participating hospital, who forwarded the electronic survey link to eligible nurses via institutional channels and professional WeChat groups. Participation was voluntary and anonymous. Ethical approval was obtained from the Capital Medical University Ethics Committee (Approval No: Z2023SY051). Electronic informed consent was obtained from all participants before they accessed the questionnaire.

### Questionnaire development

The questionnaire was developed based on the study objectives and relevant literature. Survey development involved a rigorous three-round expert review process. Specialists from the Chinese PLA General Hospital, Capital Medical University, and Peking Union Medical College reviewed the draft questionnaire with respect to overall structure, item relevance and clarity, and methodological appropriateness. Revisions were made after each round to improve wording, response options, and coherence of the instrument. Because no formal psychometric validation procedure was undertaken for the investigator-developed items, the expert review process was used to strengthen content relevance and face/content validity considerations, rather than to imply full scale validation (Polit et al., 2007; Brod et al., 2009; O'Connor, 2022).

The final questionnaire consisted of four sections. Part 1 collected demographic and occupational information, including gender, employment type, education level, monthly income, and professional title. Part 2 assessed family-related circumstances, including child-rearing status, number of children, responsibility for elder support, self-care ability of supported elders, and whether eldercare responsibilities were shared within the family. Perceived family harmony was assessed using a single investigator-developed global item asking participants to rate their satisfaction with family harmony on a 5-point Likert scale (1 = extremely satisfied to 5 = dissatisfied), with higher scores indicating poorer perceived family harmony. This variable was treated as a single-item contextual indicator rather than as a validated multidimensional construct (Fisher et al., 2016; de Boer et al., 2004).

Part 3 assessed workload characteristics, including weekly working hours, weekly working days, weekly night shifts, continuous working hours per day, frequency of ≥12-h shifts per week, requirement to perform duties beyond formal nursing responsibilities, and perceived workload level. Extra-role work was measured using a single investigator-developed yes/no item asking whether participants were required to perform duties beyond their formal nursing responsibilities. This item was treated as a direct work-context indicator rather than as a validated scale (Fisher et al., 2016; de Boer et al., 2004).

Part 4 assessed work engagement using the Utrecht Work Engagement Scale (UWES-17), which measures three dimensions: dedication, vitality, and absorption. The scale contains 17 items rated from 0 (never) to 6 (always/every day) (Keyko et al., 2016; Torabinia et al., 2017). The overall work engagement score was calculated as the mean of all 17 items, with higher scores indicating greater engagement. For each dimension, an item-average score was calculated by dividing the summed score for that dimension by the number of items. The overall work engagement score was also calculated as the mean of all 17 items. Higher scores indicate greater work engagement. For descriptive purposes, both item-average scores and total scores are reported. In the present sample, Cronbach’s alpha for the UWES-17 was 0.941.

### Sampling strategy and data collection

This study used a stratification-informed, multicenter sampling strategy rather than a fully probabilistic representative sample of all hospitals in Beijing. Hospitals were categorized according to three prespecified characteristics: hospital grade, ownership, and geographic location. Within these strata, hospitals were purposively selected to ensure diversity in organizational context and structural characteristics. A total of nine hospitals were included.

Within each participating hospital, all registered nurses listed in the personnel registry who met the eligibility criteria were invited to participate. Thus, nurse recruitment followed a census approach within the selected hospitals. To improve transparency regarding recruitment and site characteristics, Supplementary Table 1 presents the participating hospitals, including hospital grade, ownership, geographic location, and the numbers of eligible nurses invited, questionnaires returned, and valid questionnaires included. In the present study, the response rate was defined as the number of questionnaires returned divided by the number of eligible nurses invited, and the valid response rate was defined as the number of valid questionnaires included divided by the number of eligible nurses invited.

Data were collected using the SO JUMP platform. The anonymous electronic survey link was configured to allow only one submission per respondent. Before entering the questionnaire, all participants were presented with study information and provided electronic informed consent. Participation was voluntary, and respondents could withdraw at any time without penalty.

### Data management and quality control

Questionnaire responses were exported directly from the SO JUMP platform and screened before analysis. All questionnaire items were set as mandatory in the online system to prevent item-level missing data. Responses were excluded if the completion time was less than 120 s or if they showed implausible response patterns indicative of insufficient engagement (e.g., straight-lining across items). After screening, valid questionnaires were assigned unique study IDs and entered the final analytic dataset. Because completion of all items was required before submission, the final analytic dataset contained no missing questionnaire data; therefore, multiple imputation was not performed (Sterne et al., 2009).

Given the cross-sectional self-report design, potential common method bias was considered. Procedural measures included anonymous participation and structural separation of contextual/workload items from the work engagement section. Harman’s single-factor test was used only as a supplementary diagnostic (Podsakoff et al., 2003). In the exploratory factor analysis, the first unrotated factor accounted for 26.2% of the total variance, suggesting that substantial common method bias was unlikely, although it could not be fully excluded (Podsakoff et al., 2003).

### Statistical analysis

Data were analyzed using SPSS 26.0 (IBM Corp., Armonk, NY, United States). Categorical variables are presented as frequencies and percentages, and continuous variables are presented as means ± standard deviations. Work engagement was analyzed at the dimension level, including dedication, vitality, and absorption. For each dimension, the item-average score was treated as a continuous outcome. Because nurses were clustered within hospitals, generalized estimating equations (GEE) were used to account for within-hospital correlation, with hospital specified as the clustering variable (Zeger and Liang, 1986). All GEE models used a Gaussian distribution and an identity link. An exchangeable working correlation structure was specified for the primary analyses, with robust (sandwich) standard errors. Sensitivity analyses using an independence working correlation structure were also conducted, and QIC was used to compare model fit. For each dimension, univariable GEE analyses were first performed for all candidate predictors. Covariates for the multivariable GEE models were specified *a priori* using a full-model approach guided by the Job Demands-Resources framework and the aims of the study, rather than selected solely on the basis of univariable statistical significance. All prespecified covariates were entered simultaneously into each multivariable model for dedication, vitality, and absorption. Univariable analyses were retained for descriptive and exploratory purposes only. Before multivariable modeling, collinearity among predictors was assessed using tolerance statistics and variance inflation factors. Results are reported as regression coefficients (B) with 95% Wald confidence intervals (CIs) and *p*-values. A two-sided *p*-value < 0.05 was considered statistically significant.

## Results

### Demographic characteristics of nurses

A total of 2,040 eligible nurses from the nine participating hospitals were invited to participate. Of these, 2,020 returned questionnaires, yielding a response rate of 99.0%. After data screening, 2,017 valid questionnaires were included in the final analysis, corresponding to a valid response rate of 98.9% (Supplementary Table 1).

The sample was predominantly female (95.4%). Most participants were aged 35 years or younger (66.1%), married (68.1%), and had 10 years or less of nursing practice (47.0%). In terms of department, the largest proportions worked in internal medicine wards (28.5%) and surgical wards (22.6%). More than half of the participants were contract nurses (60.7%), whereas 36.9% were permanent nurses. Most nurses held a bachelor’s degree (66.1%), and 40.1% reported a monthly income of 8,001–10,000 RMB. Nurse practitioner was the most common professional title (40.2%), followed by nurse-in-charge (31.6%) and nurse (24.4%) (Table 1).

**Table 1:** Demographic characteristics of nurses in Beijing (*N* = 2017).

Variables	Frequency, *n* (%)
Gender	
Male	93 (4.6)
Female	1924 (95.4)
Age	
≤35	1,333 (66.1)
36–50	570 (28.3)
≥51	114 (5.7)
Marital status	
Unmarried	609 (30.2)
Married	1,374 (68.1)
Divorced or other	34 (1.7)
Years of practice	
≤10	948 (47.0)
11–20	761 (37.7)
21–30	199 (9.9)
≥31	109 (5.4)
Department	
Internal Medicine Ward	575 (28.5)
Surgical Ward	456 (22.6)
Emergency or Intensive Care Unit	183 (9.1)
Operating Room	99 (4.9)
Outpatient Department	206 (10.2)
Women or Children’s Ward	138 (6.8)
Specialized Department or others	360 (17.8)
Employment type	
Permanent nurse	744 (36.9)
Contract nurse	1,224 (60.7)
Others	49 (2.4)
Education	
Secondary vocational education	37 (1.8)
Higher vocational education	614 (30.4)
Bachelor’s degree	1,333 (66.1)
Master’s degree	33 (1.6)
Income per month, RMB	
≤3,500	76 (3.8)
3,501–5,000	137 (6.8)
5,001–8,000	426 (21.1)
8,001–10,000	809 (40.1)
≥10,001	569 (28.2)
Positional titles	
Nurse	493 (24.4)
Nurse practitioner	811 (40.2)
Nurse-in-charge	637 (31.6)
Vice Professor	66 (3.3)
Chief nurse	10 (0.5)
How many children are you raising?	
0	819 (40.6)
1	850 (42.1)
≥2	348 (17.3)
Educational level of children	
Preschool age	
Yes	327 (16.2)
No	871 (43.2)
Primary school	
Yes	672 (33.2)
No	526 (26.1)
Middle school and high school	
Yes	196 (9.7)
No	1,002 (49.7)
University and above	
Yes	166 (8.2)
No	1,032 (51.2)
Whether or not you need support elderly?	
Yes	1,620 (80.3)
No	397 (19.7)
Self-care ability of elderly	
Self-cared	1,686 (83.6)
Semi-self-cared	298 (14.8)
Unable to care for self	33 (1.6)
Burden sharing situation of elderly	
Need to take care of the elderly	861 (42.7)
Elderly people help you take care of your family	1,156 (57.3)
How do you feel about the level of harmony in your family?	
Level 1	966 (47.9)
Level 2	765 (37.9)
Level 3	268 (13.3)
Level 4	16 (0.8)
Level 5	2 (0.1)

### Family situation, workload and work engagement levels among nurses

Regarding family-related characteristics, 59.4% of the participants were raising at least one child, including 42.1% raising one child and 17.3% raising two or more children. Among the educational stages of children, primary school was the most frequently reported category. In addition, 80.3% of the nurses reported that they needed to support older family members, most of whom were able to care for themselves (83.6%). Nearly half of the participants rated family harmony at Level 1 (47.9%), and 57.3% reported that older family members helped share family responsibilities or childcare (Table 1).

Workload characteristics indicated substantial work demands. Only 35.2% of nurses worked 40 h or less per week, whereas 42.2% worked 41–45 h and 22.6% worked 46 h or more per week. Most participants worked 5 days or less per week (86.9%). Continuous working hours were commonly prolonged, with 52.8% reporting 5–8 h, 34.1% reporting 9–12 h, and 2.8% reporting 13 h or more per day. Night shifts were also common, with 61.8% reporting at least one night shift per week. In addition, 45.8% reported being required to perform work outside their formal duties and responsibilities. Perceived workload was predominantly moderate (60.8%) or severe/extremely severe (34.0%), while only 5.2% reported a mild workload (Table 2).

**Table 2:** Workload of nurses in Beijing (*N* = 2017).

Variables	Frequency, *n* (%)
Working hours per week	
≤40 h	709 (35.2)
41–45 h	851(42.2)
46–50 h	279 (13.8)
51–55 h	73 (3.6)
56–60 h	56 (2.8)
≥61 h	49 (2.4)
Working days per week	
≤5 d	1752 (86.9)
6 d	248 (12.3)
7 d	17 (0.8)
Night shift per week	
None	772 (38.2)
1–2	869 (43.1)
3–4	342 (17.0)
≥5	34 (1.7)
Continuous working hours per day	
≤4 h	208 (10.3)
5–8 h	1,065 (52.8)
9–12 h	688 (34.1)
≥13 h	56 (2.8)
The days that need nurses working ≥12 h per week	
None	1,052 (52.2)
1–3	865 (42.8)
≥4	100 (5.0)
Do you need to take on any other work outside of duties and responsibilities?	
Yes	924 (45.8)
No	1,093 (54.2)
What is the perceived level of workload?	
Mild	104 (5.2)
Moderate	1,226 (60.8)
Severe	628 (31.1)
Extremely severe	59 (2.9)

As shown in Table 3, the mean overall work engagement item-average score was 4.42 ± 1.25, corresponding to a total score of 74.90 ± 21.25. Among the three dimensions, dedication had the highest mean item-average score (4.72 ± 1.27), followed by vitality (4.37 ± 1.29) and absorption (4.22 ± 1.30).

**Table 3:** Descriptive statistics of Utrecht work engagement scale scores among nurses in Beijing (*N* = 2017, mean ± SD).

Items	Average score	Total score
Work engagement	4.42 ± 1.25	74.90 ± 21.25
Dedication	4.72 ± 1.27	23.58 ± 6.37
Vitality	4.37 ± 1.29	26.21 ± 7.74
Absorption	4.22 ± 1.30	25.11 ± 7.89

### Factors associated with work engagement dimensions among nurses

As shown in Table 4, poorer family harmony was the most consistent factor associated with lower work engagement across all three dimensions. Compared with Level 1 family harmony, Level 2, Level 3, and Level 4–5 harmony were all significantly associated with lower dedication, vitality, and absorption scores, with the magnitude of association generally increasing as family harmony worsened. In the multivariable models, Level 3 family harmony was associated with lower dedication (*B* = −0.788, 95% Wald CI: −0.904 to −0.673, *p* < 0.001), vitality (*B* = −0.689, 95% Wald CI: −0.767 to −0.612, *p* < 0.001), and absorption scores (*B* = −0.588, 95% Wald CI: −0.663 to −0.512, *p* < 0.001), while Level 4–5 harmony showed similarly strong negative associations with dedication (*B* = −0.729, 95% Wald CI: −0.913 to −0.545, *p* < 0.001), vitality (*B* = −1.097, 95% Wald CI: −1.386 to −0.807, *p* < 0.001), and absorption (*B* = −0.876, 95% Wald CI: −1.184 to −0.567, *p* < 0.001).

**Table 4:** Univariable and multivariable generalized estimating equation analyses of factors associated with item-average scores of work engagement dimensions among nurses.

Variables	Dedication		Vitality		Absorption	
	Multivariable		Multivariable		Multivariable	
	*B* (95% Wald CI)	*P*-value	*B* (95% Wald CI)	*P*-value	*B* (95% Wald CI)	*P*-value
Gender						
Female	Reference		Reference		Reference	
Male	0.090 (−0.181–3.62)	0.515	0.164 (−0.030–0.358)	0.097	0.192 (0.000–0.384)	0.050
Age						
≤35	Reference		Reference		Reference	
36–50	0.050 (−0.189–0.289)	0.583	0.095 (−0.142–0.332)	0.432	0.082 (−0.121–0.286)	0.428
≥51	0.539 (0.160–0.917)	0.005	0.700 (0.436–0.963)	<0.001	0.778 (0.513–1.042)	< 0.001
Marital status						
Unmarried	Reference		Reference		Reference	
Married	0.256 (0.113–0.398)	0.001	0.126 (−0.011–0.263)	0.072	0.076 (−0.068–0.220)	0.302
Divorced or other	−0.222 (−0.417 – −0.028)	0.025	−0.291 (−0.478 – −0.104)	0.002	−0.408 (−0.606 – −0.210)	0.001
Years of practice						
≤10	Reference		Reference		Reference	
11–20	0.020 (−0.081–0.122)	0.692	−0.020 (−0.180–0.121)	0.699	−0.266 (−0.670–0.139)	0.198
21–30	0.056 (−0.280–0.393)	0.742	−0.052 (−0.446–0.343)	0.797	−0.050 (−0.447–0.348)	0.807
≥31	−0.076 (−0.623–0.471)	0.786	−0.315 (−0.681 – −0.051)	0.091	0.011 (−0.150–0.171)	0.896
Department						
Internal Medicine Ward	Reference		Reference		Reference	
Surgical Ward	−0.003 (−0.155–0.148)	0.964	−0.003 (−0.167–0.160)	0.967	0.005 (−0.160–0.170)	0.953
Emergency or Intensive Care Unit	0.023 (−0.153–0.198)	0.801	−0.012 (−0.167–0.022)	0.882	−0.047 (−0.228–0.134)	0.612
Operating Room	0.584 (0.407–0.762)	<0.001	0.642 (0.407–0.877)	<0.001	0.672 (0.472–0.872)	<0.001
Outpatient Department	−0.061 (−0.283–0.161)	0.591	0.029 (−0.181–0.239)	0.788	−0.020 (−0.159–0.120)	0.780
Women or Children’s Ward	−0.002 (−0.252–0.248)	0.987	0.000 (−0.308–0.307)	0.998	0.030 (−0.252–0.313)	0.833
Specialized Department or others	−0.162 (−0.227 – −0.097)	<0.001	−0.131 (−0.198 – −0.063)	<0.001	−0.190 (−0.240 – −0.141)	<0.001
Employment type						
Permanent nurse	Reference		Reference		Reference	
Contract nurse	0.000 (−0.411–0.412)	0.999	0.092 (−0.087–0.272)	0.313	0.119 (−0.062–0.229)	0.197
Others	0.135 (−0.017–0.286)	0.081	−0.012 (−0.367–0.342)	0.945	0.014 (−0.300–0.328)	0.931
Education						
Bachelor’s degree	Reference		Reference		Reference	
Secondary vocational education	−0.578 (−0.878 – −0.277)	<0.001	−0.472 (−0.848 – −0.096)	0.014	−0.497 (−0.952–0.043)	0.032
Higher vocational education	−0.074 (−0.156–0.009)	0.079	−0.099 (−0.191 – −0.007)	0.035	−0.095 (−0.198–0.009)	0.074
Master’s degree	0.106 (−0.179–0.391)	0.465	0.208 (−0.051–0.466)	0.115	0.057 (−0.200–0.314)	0.662
Income						
≤5,000	Reference		Reference		Reference	
5,001–8,000	0.105 (−0.133–0.343)	0.387	0.097 (−0.115–0.309)	0.368	0.113 (−0.075–0.300)	0.239
8,001–10,000	0.181 (−0.035–0.398)	0.101	0.252 (0.022–0.482)	0.032	0.224 (0.032–0.416)	0.022
≥ 10,001	0.383 (0.114–0.652)	0.005	0.437 (0.242–0.632)	<0.001	0.398 (0.265–0.531)	<0.001
Positional titles						
Nurse	Reference		Reference		Reference	
Nurse practitioner	−0.132 (−0.244 – −0.020)	0.021	−0.168 (−0.280 – −0.057)	0.003	−0.150 (−0.235 – −0.065)	0.001
Nurse-in-charge	−0.143 (−0.364–0.078)	0.204	−0.158 (−0.364–0.048)	0.132	−0.121 (−0.360–0.117)	0.319
Vice professor	0.011 (−0.225–0.246)	0.929	0.020 (−0.189–0.228)	0.853	0.082 (−0.111–0.274)	0.405
Chief nurse	0.744 (0.167–1.320)	0.011	0.589 (0.285–0.894)	0.001	0.481 (0.166–0.796)	0.003
How many children are you raising?						
0	Reference		Reference		Reference	
1	−0.112 (−0.307–0.083)	0.262	0.057 (−0.102–0.217)	0.154	0.018 (−0.203–0.239)	0.874
≥2	−0.099 (−0.248–0.050)	0.194	0.074 (−0.028–0.176)	0.482	0.078 (−0.090–0.246)	0.362
Whether or not you need support elderly?						
Yes	Reference		Reference		Reference	
No	−0.083 (−0.207–0.040)	0.187	−0.073 (−0.210–0.065)	0.300	−0.113 (−0.258–0.032)	0.126
Self-care ability of elderly						
Self-cared	Reference		Reference		Reference	
Semi-self-cared	0.074 (−0.030–0.177)	0.164	0.065 (−0.054–0.184)	0.282	0.139 (−0.024–0.301)	0.094
Unable to care for self	0.281 (0.115–0.446)	<0.001	0.276 (−0.040–0.592)	0.087	0.234 (−0.192–0.660)	0.282
Burden sharing situation of elderly						
Need to take care of the elderly	Reference		Reference		Reference	
Elderly people help you	0.020 (−0.040–0.079)	0.519	0.036 (−0.040–0.113)	0.352	0.047 (−0.042–0.136)	0.298
How do you feel about the level of harmony in your family?						
Level 1	Reference		Reference		Reference	
Level 2	−0.500 (−0.608 – −0.393)	<0.001	−0.502 (−0.599 – −0.406)	<0.001	−0.459 (−0.528 – −0.389)	<0.001
Level 3	−0.788 (−0.904 – −0.673)	<0.001	−0.689 (−0.767 – −0.612)	<0.001	−0.588 (−0.663 – −0.512)	<0.001
Levels 4 and 5	−0.729 (−0.913 – −0.545)	<0.001	−1.097 (−1.386 – −0.807)	<0.001	−0.876 (−1.184 – −0.567)	<0.001
Working hours per week						
≤40 h	Reference		Reference		Reference	
41–45 h	−0.239 (−0.354 – −0.124)	<0.001	−0.179 (−0.283 – −0.075)	0.001	−0.231 (−0.358 – −0.104)	0.001
46–50 h	−0.092 (−0.231–0.046)	0.190	−0.110 (−0.229 – −0.009)	0.069	−0.133 (−0.258 – −0.009)	0.036
51–55 h	−0.142 (−0.559–0.274)	0.503	0.037 (−0.396–0.470)	0.867	−0.068 (−0.453–0.318)	0.731
56–60 h	0.217 (0.077–0.356)	0.002	0.244 (−0.017–0.504)	0.067	0.153 (−0.061–0.366)	0.161
≥61 h	0.428 (0.235–0.621)	< 0.001	0.503 (0.336–0.670)	<0.001	0.385 (0.255–0.516)	<0.001
Working days per week						
≤5 d	Reference		Reference		Reference	
≥6 d	−0.046 (−0.165–0.073)	0.452	−0.001 (−0.092–0.090)	0.984	0.050 (−0.077–0.177)	0.439
Night shift per week						
None	Reference		Reference		Reference	
1–2	−0.026 (−0.112–0.061)	0.561	−0.078 (−0.148 – −0.009)	0.028	−0.061 (−0.119 – −0.002)	0.042
3–4	−0.166 (−0.324 – −0.008)	0.039	−0.205 (−0.347 – −0.062)	0.005	−0.230 (−0.371 – −0.089)	0.001
≥5	−0.058 (−0.644–0.527)	0.845	0.040 (−0.520–0.600)	0.889	0.013 (−0.705–0.730)	0.973
Continuous working hours per day						
5–8 h	Reference		Reference		Reference	
≤4 h	0.011 (−0.142–0.165)	0.886	−0.012 (−0.142–0.117)	0.852	0.005 (−0.111–0.120)	0.939
9–12 h	0.062 (−0.077–0.202)	0.380	0.025 (−0.087–0.137)	0.657	0.035 (−0.104–0.173)	0.621
≥13 h	−0.097 (−0.375–0.182)	0.497	−0.157 (−0.505–0.190)	0.374	−0.089 (−0.376–0.198)	0.545
The days that need nurses working ≥ 12 h per week						
None	Reference		Reference		Reference	
1–3	−0.041 (−0.117–0.035)	0.288	−0.006 (−0.089–0.077)	0.888	−0.034 (−0.119–0.052)	0.428
≥4	−0.302 (−0.411 – −0.192)	<0.001	−0.246 (−0.352 – −0.140)	<0.001	−0.295 (−0.386 – −0.203)	<0.001
Do you need to take on any other work outside of duties and responsibilities?						
No	Reference		Reference		Reference	
Yes	−0.194 (−0.314 – −0.074)	0.002	−0.189 (−0.321 – −0.057)	0.005	−0.183 (−0.289 – −0.077)	<0.001
What is the perceived level of workload?						
Mild	Reference		Reference		Reference	
Moderate	−0.238 (−0.512–0.035)	0.088	−0.331 (−0.660 – −0.002)	0.048	−0.321 (−0.652–0.009)	0.057
Severe	−0.524 (−0.875 – −0.173)	0.00	−0.666 (−1.063 – −0.269)	0.001	−0.573 (−0.954 – −0.192)	0.003
Extremely severe	−1.024 (−1.432 – −0.617)	0.001	−1.098 (−1.589 – −0.608)	<0.001	−1.067 (−1.435 – −0.700)	<0.001

All categorical predictors were entered using dummy coding, and the reference category for each variable is indicated as “Reference” in the table. Each column presents results from a multivariable GEE model including the same prespecified covariates.

Workload-related factors also showed robust associations. Working 41–45 h per week was associated with lower dedication, vitality, and absorption scores than working ≤40 h per week, whereas working ≥12 h on at least 4 days per week was independently associated with lower dedication (*B* = −0.302, 95% Wald CI: −0.411 to −0.192, *p* < 0.001), vitality (*B* = −0.246, 95% Wald CI: −0.352 to −0.140, *p* < 0.001), and absorption (*B* = −0.295, 95% Wald CI: −0.386 to −0.203, *p* < 0.001). Undertaking work outside formal duties and responsibilities was likewise associated with lower dedication (*B* = −0.194, 95% Wald CI: −0.314 to −0.074, *p* = 0.002), vitality (*B* = −0.189, 95% Wald CI: −0.321 to −0.057, *p* = 0.005), and absorption scores (*B* = −0.183, 95% Wald CI: −0.289 to −0.077, *p* < 0.001). In addition, perceived workload showed an inverse pattern, with severe and extremely severe workload showing progressively larger negative coefficients across all three dimensions.

Several demographic and occupational characteristics were also independently associated with work engagement. Nurses aged ≥51 years had higher dedication, vitality, and absorption scores than those aged ≤35 years. Compared with internal medicine wards, working in the operating room was positively associated with all three dimensions, whereas working in specialized departments or other departments was negatively associated with all three. Higher monthly income, particularly ≥10,001 RMB, was consistently associated with higher scores across the three dimensions. Chief nurses also had significantly higher scores in dedication, vitality, and absorption, whereas nurse practitioners had lower adjusted scores than nurses. In addition, divorced or other marital status was associated with lower scores across all three dimensions, and male nurses showed a borderline higher absorption score than female nurses. Overall, poorer family harmony and heavier work demands were the most consistent correlates of lower work engagement, whereas older age, operating-room placement, higher income, and senior professional title were generally associated with higher work engagement.

## Discussion

This study provides a multidimensional picture of work engagement among hospital nurses in Beijing. Three findings deserve particular emphasis. First, work engagement remained relatively high overall, with dedication showing the highest mean score among the three UWES dimensions. Second, family harmony emerged as the most consistent correlate across dedication, vitality, and absorption. Third, workload-related factors were strongly and repeatedly associated with lower engagement, especially higher perceived workload, repeated long-duty days, and the requirement to undertake work outside formal duties and responsibilities. In addition, several demographic and occupational variables, including older age, departmental placement, income, and positional title, remained independently associated with one or more dimensions of engagement after adjustment. These findings are broadly consistent with contemporary nursing literature, which frames work engagement as a function of the balance between job demands and available resources rather than as a fixed personal attribute alone (BowenXue et al., 2024; Wang et al., 2021; Zhang et al., 2025; Montgomery et al., 2015; Aungsuroch et al., 2024).

Among all variables examined, family harmony showed the clearest and most consistent inverse association with work engagement. Compared with nurses reporting the highest level of family harmony, those reporting poorer family harmony had lower scores in all three dimensions. This pattern is important because it suggests that family context is not merely a background characteristic, but a substantive source of psychological resource gain or depletion. In the nursing literature, family-related resources such as family mastery, family support, and work-family enrichment have been linked to stronger engagement, whereas work-family imbalance tends to erode it (Lu et al., 2011; Zhang et al., 2021; Fukuzaki et al., 2021). In the present study, “family harmony” likely captured several interrelated elements at once, including emotional stability within the household, practical support for childcare or eldercare, and the degree to which nurses could recover psychologically outside work. In a workforce in which many participants were simultaneously managing children and responsibilities toward older family members, these family conditions are likely to be highly consequential for sustaining energy, concentration, and commitment at work.

Workload-related findings were equally striking. Perceived workload showed an inverse pattern, with severe and extremely severe workload associated with markedly lower scores across all three dimensions. Repeated extended-duty exposure was also important: working at least 12 h on four or more days per week was negatively associated with dedication, vitality, and absorption, even after adjustment for other factors. Likewise, the requirement to undertake work outside formal duties and responsibilities was consistently associated with lower engagement. These results suggest that the problem is not only how much nurses work, but also how their work is structured and whether it remains aligned with their professional role. This interpretation is compatible with prior evidence showing that workload is negatively related to nurse engagement and that illegitimate or non-core tasks can undermine occupational well-being, meaningfulness of work, and motivation (Wang et al., 2021; Montgomery et al., 2015; Kilponen et al., 2021; Pennbrant and Dåderman, 2021). From a management perspective, the present findings argue for greater attention to role protection, reduction of non-nursing tasks, and closer monitoring of sustained high-intensity scheduling patterns rather than focusing on total hours alone.

One result requires particularly cautious interpretation: after multivariable adjustment, nurses working at least 61 h per week had higher scores across all three engagement dimensions. This should not be read as evidence that very long working hours are beneficial. A more plausible interpretation is that this subgroup reflects selection and role-composition effects: highly engaged nurses, nurses in more senior or specialized positions, or nurses with stronger professional identification may be disproportionately represented among those working the longest hours. Reverse causation is also possible in a cross-sectional design, whereby engagement increases willingness to remain at work longer rather than long hours increasing engagement. This interpretation is more consistent with the wider literature, which generally links long shifts and fatigue-prone work patterns to burnout, dissatisfaction, cognitive strain, and threats to patient safety (Stimpfel et al., 2012; Gander et al., 2019). The contrast between the adverse effects of repeated ≥12-h duty days and the positive coefficient for the ≥61-h group further supports the view that weekly hours alone are an incomplete proxy for workload burden and may also reflect unmeasured differences in role, autonomy, scheduling control, or institutional context.

The associations observed for age, department, income, and positional title also merit comment. Nurses aged 51 years or older had higher scores across all three dimensions, which may reflect accumulated clinical confidence, greater role clarity, and better strategies for regulating emotional and operational demands. Higher income and senior title, especially chief nurse status, were likewise associated with higher engagement, a pattern that is plausibly linked to greater professional recognition, decision latitude, and perceived return for effort. By contrast, nurse practitioners had lower adjusted scores than staff nurses, suggesting that mid-level roles may carry substantial responsibility without commensurate resource gain in some settings. Departmental differences were also notable. Operating-room nurses showed higher engagement across all three dimensions, whereas nurses in specialized or heterogeneous other departments showed lower scores. These patterns should be interpreted cautiously, but they raise the possibility that more structured workflows, clearer task boundaries, and tighter team coordination may support engagement, while fragmented or less standardized work settings may dilute it. The finding that divorced or other marital status was associated with lower engagement across all dimensions is also consistent with the broader picture that social and relational resources matter. Finally, the borderline higher absorption score observed in male nurses should be interpreted conservatively given the small proportion of men in the sample. Contemporary studies in Chinese nurses likewise indicate that work engagement varies across personal and professional characteristics and is shaped by a combination of work-family conditions, job characteristics, and psychosocial resources (Zhang et al., 2025; Aungsuroch et al., 2024).

These findings have several practical implications. Interventions to strengthen nurse engagement should not be limited to motivational messaging or individual resilience training. The present results point more strongly toward organizational redesign. In particular, hospitals should reduce illegitimate or non-duty work, monitor recurrent extended-duty scheduling, and respond early to rising perceived workload before strain becomes entrenched. At the same time, family-supportive policies deserve greater attention. Scheduling flexibility, predictable rosters, emergency family leave arrangements, and local support for childcare or eldercare may be especially relevant in sustaining engagement in nurses with substantial family responsibilities. In other words, protecting work engagement likely requires action on both sides of the work-family interface: reducing avoidable job demands while preserving the social and organizational resources that allow nurses to remain psychologically available for their work (Aungsuroch et al., 2024; Zhang et al., 2021; Fukuzaki et al., 2021; Kilponen et al., 2021; Pennbrant and Dåderman, 2021).

Several limitations should be acknowledged. Recruitment relied on departmental nursing managers, which may have introduced selection effects. The study was cross-sectional and therefore cannot establish temporal or causal relationships. All data were self-reported, raising the possibility of reporting bias despite anonymity and procedural efforts to reduce common method variance. The study was also conducted in Beijing only, and the findings may not fully generalize to settings with different staffing structures, organizational climates, or family support systems. In addition, perceived family harmony and extra-role work were measured using single investigator-developed items rather than formally validated multi-item instruments. Although these items were included as pragmatic contextual indicators, single-item measures provide limited information on internal consistency and may not fully capture complex constructs, particularly perceived family harmony. Accordingly, associations involving these variables should be interpreted with caution. Finally, the unexpected positive association between very long weekly working hours and engagement requires replication and more detailed examination in longitudinal and mixed-methods studies.

## Conclusion

This cross-sectional study showed that nurses’ work engagement in Beijing was shaped by both family context and work-related demands. Poorer family harmony was consistently associated with lower dedication, vitality, and absorption, while heavier perceived workload, repeated long-duty days, and undertaking work outside formal duties and responsibilities were also linked to lower engagement across dimensions. By contrast, older age, higher income, senior professional title, and working in the operating room were generally associated with higher engagement. Although nurses working at least 61 h per week showed higher engagement scores, this finding should be interpreted cautiously and is more likely to reflect selection or role-related factors than a beneficial effect of excessive working hours. Overall, these findings suggest that sustaining nurse work engagement requires not only attention to individual motivation, but also organizational action to reduce avoidable workload, protect role boundaries, and strengthen family-supportive policies. Such efforts may help maintain a more resilient, stable, and effective nursing workforce.

## Data Availability

The original contributions presented in the study are included in the article/Supplementary material, further inquiries can be directed to the corresponding author.
