# Supplementary material for: Factors associated with work engagement dimensions among hospital nurses in Beijing: a cross-sectional study
Source: Front Psychol. 2026 Jun 25;17:1817637. doi: 10.3389/fpsyg.2026.1817637 (PMC13345847; doi:10.3389/fpsyg.2026.1817637)
Supplement: Supplementary file 1 [file Table_1.DOCX]

**Supplementary Table 1. Characteristics of participating hospitals and nurse recruitment by site.**

A total of 2,040 nurses responded the questionnaire, 2,020 nurses finished and 2,017 eligible nurses were included in the study. The **Valid** response rates are 98.9%.

| **Hospital code** | **Hospital grade** | **Ownership** | **Geographic location** | **Eligible nurses invited, n** | **Questionnaires returned, n** | **Valid questionnaires included, n** | **Valid Response rate, %** |
| --- | --- | --- | --- | --- | --- | --- | --- |
| Hospital A | Tertiary | Public | Urban | 420 | 419 | 419 | 99.7 |
| Hospital B | Tertiary | Public | Suburban | 385 | 380 | 379 | 98.4 |
| Hospital C | Tertiary | Private | Exurban | 310 | 309 | 309 | 99.7 |
| Hospital D | Secondary | Public | Urban | 255 | 250 | 250 | 98.0 |
| Hospital E | Secondary | Private | Suburban | 242 | 240 | 238 | 98.3 |
| Hospital F | Secondary | Public | Exurban | 182 | 180 | 180 | 98.9 |
| Hospital G | Primary | Public | Urban | 96 | 95 | 95 | 98.9 |
| Hospital H | Primary | Private | Suburban | 75 | 72 | 72 | 96.0 |
| Hospital I | Primary | Public | Exurban | 75 | 75 | 75 | 100 |
| Total |  |  |  | 2040 | 2020 | 2017 | 98.9 |

**Abbreviations: n, number.**

**Note:** Hospitals were anonymized as Hospital A–I to preserve institutional confidentiality. Eligible nurses invited were defined as registered nurses listed in the personnel registry who met the study eligibility criteria. Response rate was calculated as the number of questionnaires returned divided by the number of eligible nurses invited.

**Supplementary Table 2. Univariable generalized estimating equation analyses of factors associated with item-average scores of work engagement dimensions**

| **Variables** | **Dedication** | | **Vitality** | | **Absorption** | |
| --- | --- | --- | --- | --- | --- | --- |
|  | **Univariable** | | **Univariable** | | **Univariable** | |
|  | **B (95% Wald CI)** | ***P value*** | **B (95% Wald CI)** | ***P value*** | **B (95% Wald CI)** | ***P value*** |
| **Gender** |  |  |  |  |  |  |
| Female | Reference |  | Reference |  | Reference |  |
| Male | 0.172(0.009-0.335) | 0.038 | 0.254(0.144-0.365) | < 0.001 | 0.270(0.159-0.381) | < 0.001 |
| **Age** |  |  |  |  |  |  |
| ≤35 | Reference |  | Reference |  | Reference |  |
| 36 - 50 | 0.040(-0.145-0.224) | 0.673 | 0.114(-0.079-0.307) | 0.245 | 0.098(-0.080-0.276) | 0.280 |
| ≥ 51 | 0.438(0.167-0.710) | 0.002 | 0.508(0.245-0.772) | < 0.001 | 0.567(0.333-0.801) | < 0.001 |
| **Marital status** |  |  |  |  |  |  |
| Unmarried | Reference |  | Reference |  | Reference |  |
| Married | 0.145 (-0.038-0.329) | 0.121 | 0.169(-0.018-0.357) | 0.076 | 0.145(-0.045-0.334) | 0.135 |
| Divorced or other | -0.412(-0.576- -0.247) | < 0.001 | -0.337(-0.534- -0.140) | 0.001 | -0.420(-0.780- -0.061) | 0.022 |
| **Years of practice** |  |  |  |  |  |  |
| ≤ 10 | Reference |  | Reference |  | Reference |  |
| 11 - 20 | -0.055(-0.174-0.064) | 0.364 | -0.005(-0.137-0.126) | 0.935 | 0.024(-0.106-0.154) | 0.718 |
| 21 - 30 | 0.124(-0.063-0.310) | 0.194 | 0.193(0.004-0.382) | 0.046 | 0.141(-0.055-0.338) | 0.159 |
| ≥ 31 | 0.375(0.004-0.746) | 0.047 | 0.412(0.108-0.717) | 0.008 | 0.476(0.207-0.745) | 0.001 |
| **Department** |  |  |  |  |  |  |
| Internal Medicine Ward | Reference |  | Reference |  | Reference |  |
| Surgical Ward | 0.006(-0.186-0.197) | 0.954 | 0.003(-0.218-0.223) | 0.982 | 0.020(-0.205-0.244) | 0.862 |
| Emergency or Intensive Care Unit | 0.001(-0.193-0.194) | 0.994 | -0.045(-0.235-0.144) | 0.639 | -0.080(-0.307-0.148) | 0.492 |
| Operating Room | 0.819(0.691-0.947) | < 0.001 | 0.900(0.747-1.053) | < 0.001 | 0.911(0.749-1.073) | < 0.001 |
| Outpatient Department | 0.007(-0.162-0.175) | 0.936 | 0.107(-0.045-0.260) | 0.168 | 0.091(-0.009-0.191) | 0.075 |
| Women or Children's Ward | -0.015(-0.291-0.260) | 0.913 | -0.020(-0.337-0.297) | 0.901 | 0.010(-0.321-0.341) | 0.952 |
| Specialized Department or others | -0.102(-0.174- -0.029) | 0.006 | -0.069(-0.117- -0.021) | 0.005 | -0.121(-0.197- -0.046) | 0.002 |
| **Employment type** |  |  |  |  |  |  |
| Permanent nurse | Reference |  | Reference |  | Reference |  |
| Contract nurse | 0.035(-0.094-0.164) | 0.594 | -0.027(-0.156-0.103) | 0.687 | 0.001(-0.139-0.142) | 0.986 |
| Others | -0.247(-0.652-0.158) | 0.232 | -0.272(-0.622-0.077) | 0.127 | -0.234(-0.557-0.089) | 0.155 |
| **Education** |  |  |  |  |  |  |
| Bachelor's degree | Reference |  | Reference |  | Reference |  |
| Secondary vocational education | -0.409(-0.716- -0.102) | 0.009 | -0.270(-0.660-0.119) | 0.173 | -0.307(-0.755-0.142) | 0.180 |
| Higher vocational education | -0.014(-0.093-0.065) | 0.735 | -0.042(-0.144-0.060) | 0.422 | -0.043(-0.147-0.061) | 0.418 |
| Master's degree | 0.290(-0.045-0.625) | 0.090 | 0.460(0.131-0.789) | 0.006 | 0.261(-0.025-0.547) | 0.074 |
| **Income** |  |  |  |  |  |  |
| ≤ 5000 | Reference |  | Reference |  | Reference |  |
| 5001 - 8000 | 0.026(-0.163-0.215) | 0.789 | 0.009(-0.133-0.152) | 0.897 | 0.027(-0.137-0.191) | 0.743 |
| 8001 - 10000 | 0.169(0.006-0.333) | 0.042 | 0.213(0.070-0.356) | 0.003 | 0.197(0.045-0.348) | 0.011 |
| ≥ 10001 | 0.408 (0.217-0.599) | < 0.001 | 0.441(0.347-0.535) | < 0.001 | 0.401(0.286-0.515) | < 0.001 |
| **Positional Titles** |  |  |  |  |  |  |
| Nurse | Reference |  | Reference |  | Reference |  |
| Nurse Practitioner | -0.108(-0.250-0.035) | 0.139 | -0.109(-0.278-0.061) | 0.208 | -0.106(-0.248-0.035) | 0.139 |
| Nurse-in-charge | -0.058(-0.213-0.097) | 0.461 | 0.002(-0.187-0.191) | 0.986 | 0.013(-0.167-0.193) | 0.889 |
| Vice Professor | -0.353(-0.047-0.753) | 0.084 | 0.461(0.153-0.768) | 0.003 | 0.449(0.156-0.742) | 0.003 |
| Chief nurse | 1.281(0.483-2.079) | 0.002 | 1.226(0.753-1.700) | <0.001 | 1.066(0.610-1.522) | <0.001 |
| **How many children are you raising?** |  |  |  |  |  |  |
| 0 | Reference |  | Reference |  | Reference |  |
| 1 | 0.043(-0.175-0.261) | 0.697 | 0.131(-0.078-0.341) | 0.220 | 0.104(-0.133-0.341) | 0.388 |
| ≥ 2 | -0.035(-0.184-0.114) | 0.643 | 0.056(-0.104-0.216) | 0.495 | 0.087(-0.073-0.247) | 0.289 |
| **Whether or not you need support elderly?** |  |  |  |  |  |  |
| Yes | Reference |  | Reference |  | Reference |  |
| No | 0.008(-0.150-0.166) | 0.924 | -0.020(-0.197- 0.156) | 0.823 | -0.061(-0.252-0.130) | 0.534 |
| **Self-care ability of elderly** |  |  |  |  |  |  |
| Self-cared | Reference |  | Reference |  | Reference |  |
| Semi-self-cared | -0.044(-0.197- 0.109) | 0.572 | -0.028(-0.168- 0.112) | 0.691 | 0.068(-0.089-0.224) | 0.397 |
| Unable to care for self | 0.234(0.074-0.393) | 0.004 | 0.253(-0.005-0.510) | 0.054 | 0.227(-0.128-0.583) | 0.210 |
| **Burden sharing situation of elderly** |  |  |  |  |  |  |
| Need to take care of the elderly | Reference |  | Reference |  | Reference |  |
| Elderly people help you | 0.050(-0.033- 0.133) | 0.236 | 0.057(-0.043-0.158) | 0.264 | 0.030(-0.034-0.094) | 0.357 |
| **How do you feel about the level of harmony in your family?** |  |  |  |  |  |  |
| Level 1 | Reference |  | Reference |  | Reference |  |
| Level 2 | -0.587(-0.691- -0.482) | < 0.001 | -0.576(-0.671- -0.482) | <0.001 | -0.529(-0.629- -0.430) | <0.001 |
| Level 3 | -0.937(-1.090- -0.784) | < 0.001 | -0.829(-0.939- -0.719) | <0.001 | -0.704(-0.811- -0.598) | <0.001 |
| Level 4 & 5 | -0.702(-0.964- -0.439) | < 0.001 | -0.993(-1.279- -0.707) | <0.001 | -0.813(-1.126- 0.501) | <0.001 |
| **Working hours per week** |  |  |  |  |  |  |
| ≤ 40 h | Reference |  | Reference |  | Reference |  |
| 41 – 45 h | -0.363(-0.447- -0.280) | < 0.001 | -0.297(-0.380- -0.213) | <0.001 | -0.324(-0.418- -0.229) | <0.001 |
| 46 – 50 h | -0.328(-0.472- -0.185) | < 0.001 | -0.330(-0.457- -0.202) | <0.001 | -0.314(-0.421- -0.206) | <0.001 |
| 51 - 55 h | -0.354(-0.793-0.084) | 0.113 | -0.173(-0.689-0.344) | 0.513 | -0.246(-0.705- 0.213) | 0.293 |
| 56 – 60 h | -0.101(-0.257-0.055) | 0.204 | -0.057(-0.230-0.116) | 0.522 | -0.100(-0.246- 0.045) | 0.177 |
| ≥ 61 h | -0.007(-0.182-0.168) | 0.935 | 0.091(-0.047-0.230) | 0.196 | 0.014(-0.053- 0.081) | 0.674 |
| **Working days per week** |  |  |  |  |  |  |
| ≤5 d | Reference |  | Reference |  | Reference |  |
| ≥ 6 d | -0.253(-0.388- -0.119) | <0.001 | -0.192(-0.321- -0.064) | 0.003 | -0.145(-0.296- 0.006) | 0.060 |
| **Night shift per week** |  |  |  |  |  |  |
| None | Reference |  | Reference |  | Reference |  |
| 1 - 2 | -0.077(-0.182- 0.027) | 0.146 | -0.143(-0.214- -0.071) | <0.001 | -0.119(-0.216- -0.021) | 0.017 |
| 3 - 4 | -0.318(-0.459- -0.178) | <0.001 | -0.391(-0.501- -0.280) | <0.001 | -0.379(-0.529- -0.265) | <0.001 |
| ≥ 5 | -0.386(-0.859-0.088) | 0.111 | -0.275(-0.768-0.218) | 0.274 | -0.293(-0.959-0.374) | 0.389 |
| **Continuous working hours per day** |  |  |  |  |  |  |
| 5 – 8 h | Reference |  | Reference |  | Reference |  |
| ≤ 4 h | 0.136(-0.043-0.315) | 0.137 | 0.124(-0.059-0.306) | 0.183 | 0.131(-0.003-0.266) | 0.056 |
| 9 – 12 h | -0.095(-0.244-0.054) | 0.212 | -0.120(-0.258-0.017) | 0.086 | -0.114(-0.270-0.041) | 0.150 |
| ≥ 13 h | -0.512(-0.828- -0.196) | 0.002 | -0.561(-0.920- -0.202) | 0.002 | -0.533(-0.852- -0.213) | 0.001 |
| **The days that need nurses working ≥ 12 h per week** |  |  |  |  |  |  |
| None | Reference |  | Reference |  | Reference |  |
| 1 – 3 | -0.624(-0.730- -0.519) | <0.001 | -0.213(-0.287- -0.139) | <0.001 | -0.221(-0.291- -0.151) | <0.001 |
| ≥ 4 | -0.220(-0.298- -0.141) | <0.001 | -0.599(-0.666- -0.533) | <0.001 | -0.614(-0.692- -0.537) | <0.001 |
| **Do you need to take on any other work outside of duties and responsibilities?** |  |  |  |  |  |  |
| No | Reference |  | Reference |  | Reference |  |
| Yes | -0.279(-0.369- -0.190) | <0.001 | -0.259(-0.355- -0.164) | <0.001 | -0.248(-0.321- -0.175) | <0.001 |
| **What is the perceived level of workload?** |  |  |  |  |  |  |
| Mild | Reference |  | Reference |  | Reference |  |
| Moderate | -0.472(-0.807- -0.137) | 0.006 | -0.535(-0.890- -0.181) | 0.003 | -0.538(-0.891- -0.185) | 0.003 |
| Severe | -0.834(-1.177- -0.491) | <0.001 | -0.935(-1.311- -0.559) | <0.001 | -0.849(-1.197- -0.501) | <0.001 |
| Extremely severe | -1.373(-1.688- -1.058) | <0.001 | -1.399(-1.793- -1.005) | <0.001 | -1.386(-1.650- -1.121) | <0.001 |
